# Altered spontaneous brain activity and functional connectivity in persistent postural-perceptual dizziness

**DOI:** 10.3389/fneur.2026.1841621

**Published:** 2026-07-15

**Authors:** Jinghan Qin, Weitao Wang, Liya Shen, Yaru Liu, Tiancan Jin, Juan Wang, Daopei Zhang, Huailiang Zhang

**Affiliations:** 1 Encephalopathy Department, The First Affiliated Hospital of Henan University of Chinese Medicine, Zhengzhou, China; 2 Henan Provincial Collaborative Innovation Center for the Prevention and Treatment of Major Diseases by Integrative Medicine, The First Affiliated Hospital of Henan University of Chinese Medicine, Zhengzhou, Henan, China; 3 Henan Provincial Vertigo Disease Diagnosis and Treatment Center, The First Affiliated Hospital of Henan University of Chinese Medicine, Zhengzhou, Henan, China

**Keywords:** default mode network, persistent postural-perceptual dizziness, resting-state functional magnetic resonance imaging, salience network, vestibular–cerebellar network, visuospatial processing

## Abstract

**Background and purpose:**

Persistent postural-perceptual dizziness (PPPD) is a chronic functional vestibular disorder characterized by persistent dizziness or postural instability that is exacerbated by upright posture, motion, and complex visual stimuli. Although neuroimaging studies suggest altered visual, vestibular, and emotional processing in PPPD, comprehensive resting-state functional magnetic resonance imaging evidence remains limited. This study investigated spontaneous brain activity and functional connectivity alterations in patients with PPPD.

**Methods:**

Twenty-one patients with PPPD and 23 healthy controls underwent 3.0 Tesla resting-state functional magnetic resonance imaging. Regional spontaneous brain activity and local synchronization were assessed using low-frequency fluctuation and regional homogeneity measures. Seed-based functional connectivity analyses were performed using the left insula, right anterior cingulate cortex, and left precuneus as seed regions. Between-group differences were tested with age and sex as covariates, and exploratory clinical–imaging correlation analyses were conducted in the patient group.

**Results:**

Patients with PPPD showed altered spontaneous activity and local synchronization in the cerebellum, occipital and temporal cortices, frontal and parietal regions, anterior cingulate cortex, insula, precuneus, and motor-related areas. Functional connectivity analyses revealed abnormal connections among the insula, caudate nucleus, prefrontal cortex, supplementary motor area, anterior and posterior cingulate cortices, precuneus, fusiform gyrus, and superior temporal gyrus. Clinical–imaging correlation analyses further showed that disease duration, dizziness handicap, anxiety severity, and cognitive performance were associated with functional changes in visual, cerebellar, supplementary motor, superior parietal, cuneus, and precuneus regions.

**Conclusion:**

These findings indicate that PPPD involves distributed functional abnormalities in brain regions responsible for vestibular and postural integration, visual–spatial processing, emotional and salience monitoring, motor regulation, and self-referential cognitive processing. Abnormalities in these regions may contribute to persistent dizziness, postural instability, visual dependence, heightened symptom vigilance, anxiety-related symptom amplification, and insufficient postural–motor compensation. Thus, PPPD may be better understood as a distributed network disorder involving disrupted visual–vestibular–postural integration and maladaptive functional reorganization.

## 1 Introduction

Persistent postural-perceptual dizziness (PPPD) is a chronic balance disorder that has been formally defined in recent years and is now recognized as one of the most common chronic vestibular syndromes (1, 2). It is characterized as a “functional” dizziness, due to the absence of overt structural central or peripheral neural lesions, with core features including persistent subjective unsteadiness and/or dizziness. that is markedly exacerbated by upright posture, active or passive motion, and exposure to complex visual environments (3). Accumulating evidence suggests that patients with PPPD often exhibit elevated levels of neuroticism and anxiety, which may play a critical role in the development and perpetuation of the disorder (4, 5). In addition, these patients typically demonstrate heightened bodily vigilance, particularly toward dizziness-related sensations, a feature that is not observed in healthy individuals or in patients who have recovered from vestibular disorders (4). Such psychological factors may further amplify negative symptom perception, thereby contributing to impaired quality of life. Given that PPPD is a relatively newly defined condition, no widely accepted standardized treatment has yet been established. Current clinical management mainly includes pharmacological interventions (e.g., anxiolytics and antidepressants) and vestibular rehabilitation; however, evidence regarding their efficacy and safety remains limited (6, 7).

Current opinion suggests that PPPD may be triggered by an acute vertigo episode or psychological stress. During the recovery phase, due to anxiety and excessive attention to bodily symptoms, individuals fail to abandon these transient adaptive strategies and instead consolidate them into a persistent maladaptive pattern (8). However, the underlying mechanism remains unclear. Recent studies, which utilize neuroimaging techniques such as magnetic resonance imaging (MRI) and single-photon emission computed tomography (SPECT), have deepened our understanding of the underlying mechanisms of PPPD. Studies have shown that patients with PPPD show notable reductions in gray matter volume, metabolic activity in the visual cortex, vestibular areas, and limbic system, along with diminished cerebral blood flow (9, 10). These alterations suggest structural and functional impairments in brain regions involved in emotional regulation and visual–vestibular integration. In addition, multiple studies based on brain network analysis have revealed abnormal neural circuits in multisensory vestibular cortices and sensorimotor integration regions in patients with PPPD, together with altered topological properties of brain networks, such as nodal efficiency and connectivity strength; notably, these changes are significantly correlated with Dizziness Handicap Inventory (DHI) scores (11). Although previous studies have made important contributions to our understanding of the possible mechanisms of PPPD, the existing literature remains limited and the assessment indices employed are still not comprehensive enough. Therefore, the present study aimed to investigate regional brain functional alterations in patients with PPPD compared with healthy controls by using regional brain activity measures, including amplitude of low-frequency fluctuation (ALFF), fractional amplitude of low-frequency fluctuation (fALFF), regional homogeneity (ReHo), and seed-based functional connectivity (FC) analysis, so as to further explore the potential mechanisms involved.

## 2 Methods

### 2.1 Participants

This study was a cross-sectional case-control study. From January 2025 to January 2026, 21 patients who visited the First Affiliated Hospital of Henan University of Chinese Medicine and met the diagnostic criteria for PPPD were consecutively enrolled. Meanwhile, 23 healthy controls (HCs) were also recruited. All participants were right-handed and provided written informed consent before enrollment. Demographic data, including sex, age, and education level, were collected from both PPPD patients and HCs using standardized questionnaires. The Self-Rating Anxiety Scale (SAS) and Self-Rating Depression Scale (SDS) were also administered to both groups. Disease-related clinical information and vestibular/cognitive assessments, including duration of dizziness, symptom aggravating factors, Dizziness Handicap Inventory total score (DHI), Montreal Cognitive Assessment (MoCA), Timed Up and Go test (TUG), and Visual Analog Scale for Vertigo (V-VAS), were collected only from patients with PPPD. This study was approved by the Ethics Committee of the First Affiliated Hospital of Henan University of Chinese Medicine (approval number: 2023HL-146) and was conducted in accordance with the Declaration of Helsinki.

### 2.2 Inclusion and exclusion criteria

The diagnosis of PPPD was based on the diagnostic criteria established by the Bárány Society in 2017 (1). Specifically, all patients with PPPD were required to meet the following five criteria simultaneously: (A) the presence of one or more core vestibular symptoms, including dizziness, unsteadiness, or non-spinning vertigo, occurring on most days for 3 months or longer; symptoms usually last for hours and may fluctuate, but do not need to be present continuously throughout the day; (B) persistent symptoms may occur without specific provocation but are exacerbated by upright posture, active or passive motion, and exposure to moving visual stimuli or complex visual patterns; all three categories of aggravating factors must be identifiable in the clinical history, although their severity does not need to be identical; (C) symptoms are precipitated by conditions or events that can cause vertigo, unsteadiness, dizziness, or balance problems, including acute, episodic, or chronic vestibular syndromes, other neurological or medical illnesses, or psychological distress; after an acute or episodic precipitant, symptoms may gradually evolve into a persistent course as the precipitating condition resolves, whereas symptoms associated with chronic precipitants may develop slowly and gradually worsen; (D) symptoms cause significant distress or functional impairment; and (E) the symptoms are not better accounted for by another disease or disorder.

The exclusion criteria were as follows: (1) comorbid definite vestibular disorders, such as Ménière's disease, benign paroxysmal positional vertigo, vestibular paroxysmia, and migraine with brainstem aura; (2) dizziness caused by central nervous system diseases, as indicated by cranial magnetic resonance imaging or other auxiliary examinations, such as cerebral infarction, cerebral hemorrhage, or intracranial space-occupying lesions; (3) severe cervical spondylosis, severe cardiac, pulmonary, hepatic, or renal dysfunction, or other conditions that prevented cooperation with the examination; (4) contraindications to magnetic resonance imaging or claustrophobia; (5) pregnancy or lactation; and (6) participation in, or current participation in, other clinical trials within the previous 3 months.

At enrollment, healthy controls had no subjective symptoms of dizziness, vertigo, unsteadiness, or headache, no history of acute or chronic dizziness, vertigo, or headache, and were not regularly taking medications that might affect central nervous system or vestibular function. The same exclusion criteria related to central nervous system diseases, severe physical illnesses, and contraindications to magnetic resonance imaging were also applied to the healthy controls.

None of the patients with PPPD had received routine prophylactic treatment before enrollment, and none had taken medications for dizziness or drugs that might affect vestibular function within 24 h before the imaging assessment.

### 2.3 Image acquisition

All imaging data in the present study were acquired in the standardized MRI scanning suite of the Department of Radiology at the First Affiliated Hospital of Henan University of Chinese Medicine, using a 3.0T Ingenia CX MRI system (Philips Healthcare, Netherlands) equipped with a 16-channel head–neck coil. Participants were positioned in a standard supine posture, with head alignment adjusted using a laser positioning system. They were instructed to remain relaxed, keep their eyes closed, and stay awake throughout the scanning procedure. To ensure both image quality and participant safety, dual protective measures were implemented: auditory protection was provided using noise-reducing earplugs, and head motion was minimized using foam padding for stabilization.

All scans were performed in a head-first position, with the central alignment referenced to the midline of the forehead, following a standardized acquisition protocol. Structural images were acquired in the sagittal plane using a three-dimensional gradient-echo T1-weighted imaging sequence with the following parameters: 175 sagittal slices; repetition time (TR) = 7,500 ms; echo time (TE) = 3.70 ms; flip angle = 8°; field of view (FOV) = 200 mm × 240 mm × 256 mm;matrix size = 200 × 227; slice thickness = 2.0 mm; inter-slice gap = 0 mm; voxel size = 1.0 mm × 1.0 mm × 2.0 mm; and number of signal averages (NSA) = 1.

Resting-state functional MRI data were acquired using an echo-planar imaging (EPI) sequence with the following parameters: TR = 2,000 ms; TE = 30 ms; flip angle = 90°; FOV = 224 mm × 224 mm × 167 mm; and voxel size = 3.5 mm × 3.5 mm × 3.5 mm. A total of 240 volumes were collected over a scan duration of 8 min and 6 s. All participants remained awake during scanning, and no significant discomfort was reported during or after the procedure.

### 2.4 MRI data preprocessing

All resting-state functional magnetic resonance imaging (rs-fMRI) data were preprocessed using MATLAB (MathWorks, Natick, MA, USA), in combination with Statistical Parametric Mapping 12 (SPM12; Wellcome Centre for Human Neuroimaging, University College London, UK; http://www.fil.ion.ucl.ac.uk/spm/) and the Data Processing & Analysis for Brain Imaging (DPABI) toolbox (12). Prior to preprocessing, the raw Digital Imaging and Communications in Medicine (DICOM) files of both functional and structural images were converted into Neuroimaging Informatics Technology Initiative (NIfTI) format using the dcm2niix software. This conversion was performed to ensure compatibility with subsequent neuroimaging analysis software and to facilitate visual inspection and quality control.

The preprocessing pipeline included the following steps. First, the first 10 timepoints were removed to reduce the influence of initial scanner instability and participant adaptation. This step helps minimize artifacts related to non-steady-state magnetization and early head movement. Second, slice timing correction was performed to correct for differences in acquisition time among slices within each functional volume. Because the functional images were acquired using an interleaved sequence, with odd slices acquired first, all slices were temporally aligned to a common reference time point using interpolation. Third, head motion correction, also known as realignment, was conducted by rigidly aligning all functional volumes to the mean functional image. Participants with head motion exceeding 3 mm of translation in any direction or 3° of rotation around any axis were excluded from further analysis.

Fourth, spatial normalization was performed to transform each participant's brain images into a standard anatomical space, allowing voxel-wise comparisons across participants. This process involved two steps. The individual T1-weighted structural image was first co-registered to the mean blood oxygenation level-dependent (BOLD) functional image. The co-registered structural image was then segmented into gray matter, white matter, and cerebrospinal fluid. Based on this segmentation, nonlinear transformation parameters were estimated to map individual images into the Montreal Neurological Institute (MNI) standard template space. These transformation parameters were subsequently applied to the motion-corrected functional images, which were resampled to a voxel size of 3 × 3 × 3 mm^3^. Here, a voxel refers to the three-dimensional unit of an MRI image.

Fifth, spatial smoothing was performed using a Gaussian kernel with a full width at half maximum (FWHM) of 6 × 6 × 6 mm^3^. Spatial smoothing reduces local noise, improves the signal-to-noise ratio, and helps meet the assumptions of subsequent group-level statistical analyses. Sixth, linear detrending was applied to remove slow signal drifts that may arise from scanner instability, scanner heating, or physiological fluctuations, such as cardiac and respiratory effects. Seventh, nuisance covariate regression was conducted using a linear model to remove non-neuronal confounding signals. The nuisance covariates included signals from white matter and cerebrospinal fluid, as well as the 24 Friston head motion parameters. The 24-parameter motion model includes the six standard head motion parameters, their temporal derivatives, and the squared terms of these parameters, thereby providing a more comprehensive correction for motion-related artifacts.

Before calculating ALFF, fALFF, and ReHo, all datasets underwent this standardized preprocessing pipeline. ALFF and fALFF were computed within the frequency band of 0.01–0.08 Hz for whole-brain analyses. Following preprocessing, seed-based FC analysis was performed for predefined regions of interest (ROIs).

### 2.5 Statistical analysis

SPSS version 25.0 software (IBM, Chicago, IL, USA) was used to evaluate differences in baseline characteristics between the persistent postural-perceptual dizziness group and the healthy control group. Quantitative variables were compared between the two groups using independent-samples *t*-tests, whereas categorical variables were compared using chi-square tests. A *P* value < 0.05 was considered statistically significant.

MATLAB (MathWorks, Natick, MA, USA), in combination with SPM12 (Wellcome Centre for Human Neuroimaging, University College London, London, UK), was used to perform group-level statistical analyses of amplitude of low-frequency fluctuation (ALFF), fractional amplitude of low-frequency fluctuation (fALFF), regional homogeneity (ReHo), and seed-based functional connectivity (FC) metrics. Comparisons between the PPPD group and the healthy control group were conducted using independent-samples *t*-tests, with covariates such as age and sex corrected where applicable.

In the present study, most imaging analyses adopted a voxel-level threshold of *P* < 0.01 and a minimum cluster size of >10 voxels, including the ReHo, ALFF, and fALFF analyses, as well as the functional connectivity analyses based on the left insula and left precuneus seed regions. This thresholding strategy was intended to improve the spatial specificity of voxel-level analyses and reduce the risk of false-positive findings.

Only the functional connectivity analysis based on the right anterior cingulate cortex (ACC) seed region adopted a voxel-level threshold of *P* < 0.05, combined with cluster-level family-wise error (FWE) correction (cluster-level *P* < 0.05, FWE corrected). This analysis was an exploratory seed-based FC analysis, primarily aimed at observing potential long-range functional connectivity alterations in salience/emotion regulation-related networks in patients with PPPD. Considering that PPPD may involve relatively extensive brain network reorganization, and that a stringent voxel-level threshold may reduce the sensitivity for detecting long-range connectivity changes, a relatively sensitive voxel-level threshold was adopted in this exploratory analysis, while cluster-level FWE correction was applied to control for multiple comparisons. All significant brain regions were localized using the automated anatomical labeling (AAL) atlas, and the results were overlaid onto the MNI152 template using xjView and MRIcroGL software.

In addition, to further evaluate the relationship between clinical heterogeneity and brain functional abnormalities in patients with PPPD, exploratory clinical-imaging correlation analyses were performed in 21 patients with PPPD. Specifically, imaging metric values for each PPPD patient were matched individually with the corresponding clinical indicators, and correlations were analyzed between imaging metrics and disease duration, DHI scores, SAS scores, SDS scores, and MoCA scores. Prior to the analysis, normality tests were performed for all variables. Pearson correlation analysis was used for variables that conformed to a normal distribution, whereas Spearman rank correlation analysis was used for variables that did not conform to a normal distribution. Statistical significance was set at *P* < 0.05.

## 3 Results

### 3.1 Clinical data

The healthy control group consisted of 14 males and nine females, with a mean age of 44.35 ± 16.96 years. The persistent postural-perceptual dizziness group included 14 males and seven females, with a mean age of 50.62 ± 14.36 years. All participants were right-handed. There were no significant differences between the two groups in age or sex distribution, indicating that the two groups were generally comparable in demographic characteristics.

For the Dizziness Handicap Inventory, the total score was used in the present analysis rather than the physical, emotional, or functional subscale scores. In patients with persistent postural-perceptual dizziness, the mean Dizziness Handicap Inventory total score was 35.24 ± 17.38. Compared with healthy controls, patients with persistent postural-perceptual dizziness showed significantly higher Self-Rating Anxiety Scale scores (48.90 ± 9.86 vs. 37.43 ± 6.35, P < 0.001) and Self-Rating Depression Scale scores (49.62 ± 11.00 vs. 39.26 ± 6.49, *P* < 0.001). In addition, the mean Montreal Cognitive Assessment score was 22.43 ± 5.69, the mean Timed Up and Go test score was 1.24 ± 0.44, and the mean Visual Vertigo Analog Scale score was 4.57 ± 1.40 in the persistent postural-perceptual dizziness group.

Regarding symptom characteristics, all patients with persistent postural-perceptual dizziness reported dizziness and non-spinning vertigo, and all patients reported dizziness, unsteadiness, and non-spinning vertigo. The most common exacerbating factor was exposure to complex visual environments, followed by active or passive motion and upright posture. The demographic and clinical characteristics of the two groups are summarized in Table 1, and the distributions of core vestibular symptoms and exacerbating factors in the persistent postural-perceptual dizziness group are summarized in Table 2.

**Table 1:** Demographic and clinical characteristics of the persistent postural-perceptual dizziness and healthy control groups.

Variable	PPPD group	HC group	Statistical value	*P* value
Age, years	50.62 ± 14.36	44.35 ± 16.96	*t* = 1.317	0.195
Sex, male/female	14/7	14/9	χ^2^ = 0.159	0.690
Dizziness duration, months	57.62 ± 76.37	-	-	
DHI total score	35.24 ± 17.38	-	-	
SAS score	48.90 ± 9.86	37.43 ± 6.35	*t* = 4.627	<0.001
SDS score	49.62 ± 11.00	39.26 ± 6.49	*t* = 3.846	<0.001
MoCA score	22.43 ± 5.69	-	-	
Timed up and go test score	1.24 ± 0.44	-	-	
Visual Vertigo Analog scale score	4.57 ± 1.40	-	-	

Values are presented as mean ± standard deviation or number of participants. Age, Self-Rating Anxiety Scale scores, and Self-Rating Depression Scale scores were compared using independent-samples *t*-tests. Sex distribution was compared using the chi-square test. The Dizziness Handicap Inventory value represents the total score rather than the physical, emotional, or functional subscale scores. Dizziness duration, Dizziness Handicap Inventory, and Visual Vertigo Analog Scale were not applicable to healthy controls because they had no dizziness, vertigo, or headache symptoms at enrollment. Montreal Cognitive Assessment and Timed Up and Go test data were not assessed in the healthy control group. PPPD, persistent postural-perceptual dizziness; HC, healthy control; DHI, total score of the Dizziness Handicap Inventory; SAS, Self-Rating Anxiety Scale; SDS, Self-Rating Depression Scale; MoCA, Montreal Cognitive Assessment; TUG, Timed Up and Go test; V-VAS, Visual Vertigo Analog Scale.

**Table 2:** Clinical symptom characteristics of patients with persistent postural-perceptual dizziness.

Category	Clinical characteristic	*n*/*N* (%)
Core vestibular symptoms	Dizziness	21/21 (100.0%)
Core vestibular symptoms	Unsteadiness	21/21 (100.0%)
Core vestibular symptoms	Non-spinning vertigo	21/21 (100.0%)
Exacerbating factors	Upright posture	6/21 (28.6%)
Exacerbating factors	Active or passive motion	16/21 (76.2%)
Exacerbating factors	Complex visual environment	19/21 (90.5%)

Values are presented as *n*/*N* (%), where *N* represents the total number of patients with PPPD. Symptoms and exacerbating factors were summarized based on patients' clinical records and self-reported symptom characteristics.

### 3.2 Regional homogeneity (ReHo) results

**Figure 1:**
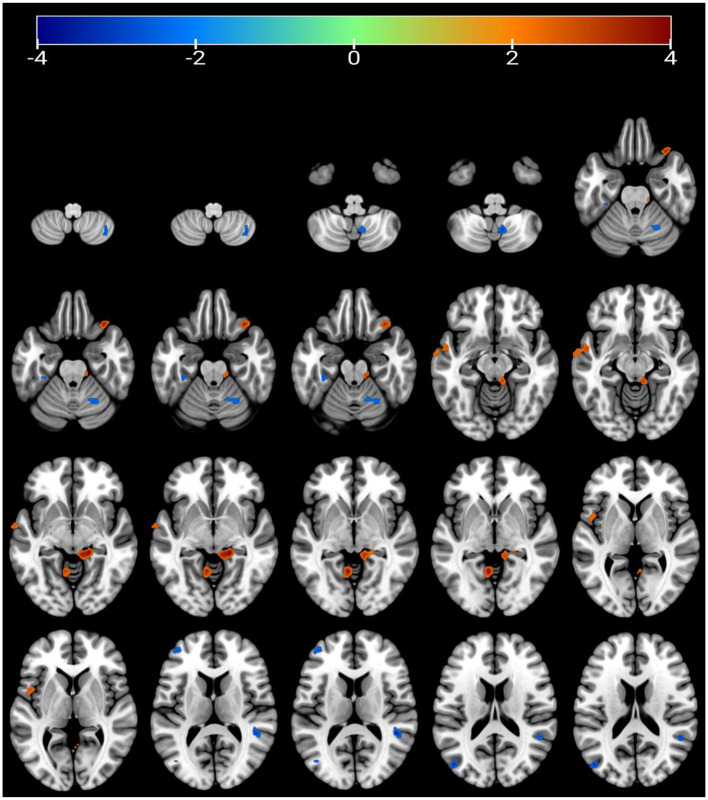
Brain regions showing global ReHo differences between the PPPD and HC groups (Red–yellow regions indicate areas with positive differences in voxel-wise *t*-test comparisons between the two groups, whereas blue–green regions indicate areas with negative differences. The background image is based on the MNI152 template. The color bar represents *T* values, with larger absolute values indicating more significant differences).

Compared with the HC group, decreased ReHo values in patients with PPPD were primarily observed in the cerebellar posterior lobe, temporal lobe, frontal lobe, and parietal regions. Specifically, reductions were found in the right cerebellar posterior lobe (lobules VI, VII, and IX), left fusiform gyrus and middle temporal gyrus, right superior temporal gyrus, left middle frontal gyrus, as well as the right superior frontal gyrus, middle frontal gyrus, medial superior frontal gyrus, and supramarginal gyrus (see Table 3, Figure 1).

**Table 3:** Results of two-sample *t*-tests for regional homogeneity between healthy controls and patients with persistent postural-perceptual dizziness (voxel *P* < 0.01, cluster size > 10).

Peak brain regions	Peak coordinates			*T* value	Cluster size
	*X*	*Y*	*Z*		
Cerebelum_8_R (aal)	36	−63	−54	3.1993	16
Cerebelum_9_R (aal)	9	−60	−45	3.3638	35
Cerebelum_6_R (aal)	15	−57	−21	2.6932	19
Fusiform_L (aal)	−33	−33	−21	3.193	12
Frontal_Mid_L (aal)	−39	48	12	3.1097	11
Temporal_Sup_R (aal)	45	−42	12	3.8155	29
Temporal_Mid_L (aal)	−45	−75	18	3.7062	14
SupraMarginal_R (aal)	51	−18	24	3.2383	19
Frontal_Sup_Medial_R (aal)	3	33	45	4.3585	26
Frontal_Mid_R (aal)	36	12	42	3.2483	11
Frontal_Sup_R (aal)	18	30	39	3.2447	11
Frontal_Inf_Orb_R (aal)	33	27	−24	3.708	29
ParaHippocampal_R (aal)	18	−36	−6	4.4235	63
Temporal_Sup_L (aal)	−54	−3	−12	2.9866	30
Cerebelum_4_5_L (aal)	−6	−54	−3	4.3836	39
Insula_L (aal)	−45	6	3	3.1689	11
Cuneus_L (aal)	0	−93	24	3.1748	11
Precentral_R (aal)	66	9	27	3.1046	13
Precuneus_L (aal)	−6	−42	63	3.9867	33
Precuneus_L (aal)	−12	−63	69	3.0117	13
Supp_Motor_Area_R (aal)	12	0	69	3.4741	17
Paracentral_Lobule_L (aal)	−6	−30	78	2.6498	14

All refers to the automated anatomical labeling atlas, which was used to define the brain region labels. Peak coordinates are reported in Montreal Neurological Institute (MNI) space. *X, Y*, and *Z* indicate the three coordinate axes in standard space. L, left; R, right.

In contrast, increased ReHo values were mainly distributed in the frontal lobe, temporal lobe, and parts of the parieto-occipital regions. These increases were observed in the right orbital part of the inferior frontal gyrus, precentral gyrus, and supplementary motor area, the left paracentral lobule and superior temporal gyrus, the right parahippocampal gyrus, as well as the left insula, cuneus, precuneus, and cerebellar lobules IV–V (see Table 3, Figure 1).

### 3.3 Amplitude of low-frequency fluctuation (ALFF) results

**Figure 2:**
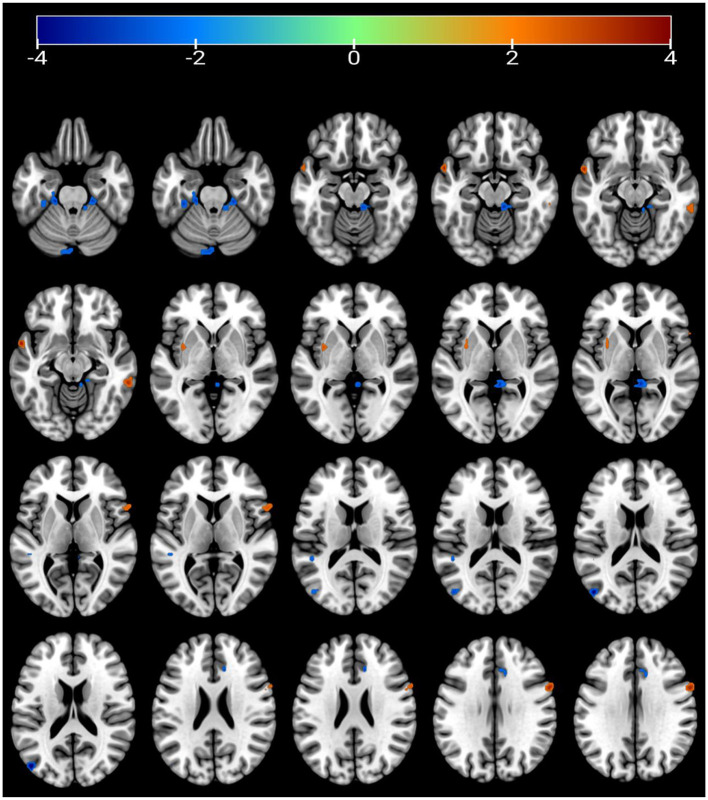
Brain regions showing global ALFF differences between the PPPD and HC groups (Red–yellow regions indicate areas with positive differences in voxel-wise *t*-test comparisons between the two groups, whereas blue–green regions indicate areas with negative differences. The background image is based on the MNI152 template. The color bar represents *T* values, with larger absolute values indicating more significant differences).

Compared with the HC group, decreased ALFF values in patients with PPPD were mainly observed in the cerebellum, occipital lobe, temporal lobe, and anterior cingulate cortex. Specifically, reductions were found in the left cerebellar lobules IV–V and Crus II, right cerebellar lobules IV–V, right lingual gyrus, left superior temporal gyrus, left middle occipital gyrus, and right anterior cingulate cortex (see Table 4, Figure 2).

**Table 4:** Results of two-sample *t*-tests for amplitude of low-frequency fluctuation between healthy controls and patients with persistent postural-perceptual dizziness (voxel *P* < 0.01, cluster size > 10).

Peak brain regions	Peak coordinates			*T* value	Cluster size
	*X*	*Y*	*Z*		
Cerebelum_4_5_L (aal)	−18	−30	−24	3.729	29
Cerebelum_Crus2_L (aal)	−3	−84	−24	3.1219	21
Cerebelum_4_5_R (aal)	21	−30	−24	3.4871	36
Lingual_R (aal)	6	−39	3	3.8014	15
Temporal_Sup_L (aal)	−45	−42	15	3.3227	13
Occipital_Mid_L (aal)	−45	−78	18	5.4662	16
Cingulum_Ant_R (aal)	12	27	24	3.7027	15
Temporal_Pole_Sup_L (aal)	−54	6	−15	4.0232	15
Temporal_Mid_R (aal)	60	−39	−12	3.4786	14
Putamen_L (aal)	−33	0	0	3.2732	10
Frontal_Inf_Oper_R (aal)	60	15	6	2.8816	14
Precentral_R (aal)	60	6	30	3.5346	23
Parietal_Inf_R (aal)	45	−42	54	4.0315	45
Precuneus_L (aal)	−6	−42	63	3.3712	12
Parietal_Sup_R (aal)	21	−63	63	4.4026	24

All refers to the automated anatomical labeling atlas, which was used to define the brain region labels. Peak coordinates are reported in Montreal Neurological Institute (MNI) space. *X, Y*, and *Z* indicate the three coordinate axes in standard space. L, left; R, right.

In contrast, increased ALFF values were primarily distributed in the temporal, frontal, and parietal lobes, as well as the basal ganglia. These increases involved the left temporal pole, right middle temporal gyrus, left putamen, right opercular part of the inferior frontal gyrus and precentral gyrus, as well as bilateral parietal regions, including the right inferior and superior parietal lobules and the left precuneus (see Table 4, Figure 2).

### 3.4 Fractional amplitude of low-frequency fluctuation (fALFF) results

**Figure 3:**
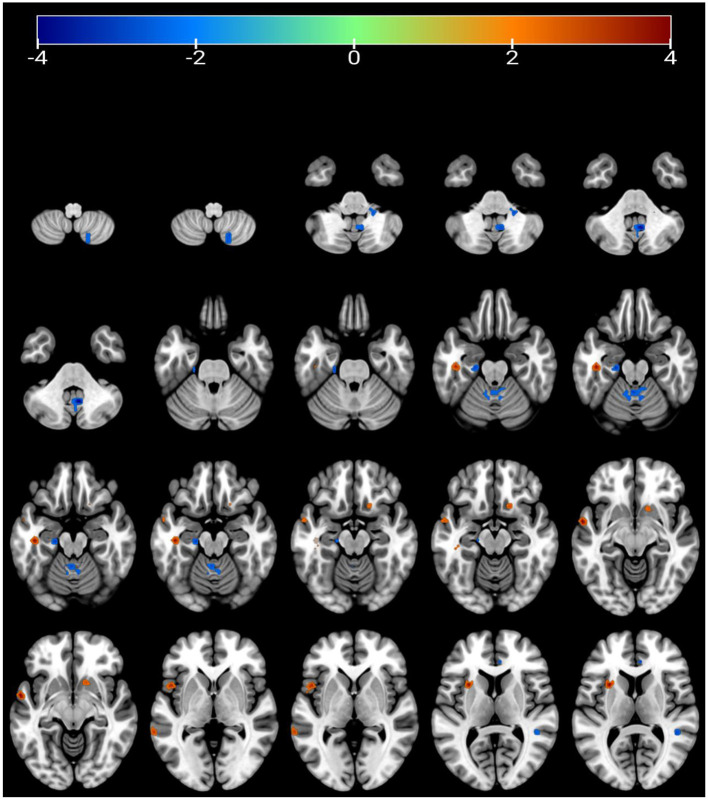
Brain regions showing global fALFF differences between the PPPD and HC groups (Red–yellow regions indicate areas with positive differences in voxel-wise *t*-test comparisons between the two groups, whereas blue–green regions indicate areas with negative differences. The background image is based on the MNI152 template. The color bar represents *T* values, with larger absolute values indicating more significant differences).

Compared with the HC group, decreased fALFF values in patients with PPPD were mainly observed in the cerebellum, temporal lobe, occipital lobe, anterior cingulate cortex, and basal ganglia. Specifically, reductions were found in the left cerebellar lobule III, right cerebellar lobules VII and X, vermis IX, left parahippocampal gyrus, right superior temporal gyrus, right anterior cingulate cortex, left middle occipital gyrus, and left caudate nucleus (see Table 5, Figure 3).

**Table 5:** Results of two-sample *t*-tests for fractional amplitude of low-frequency fluctuation between healthy controls and patients with persistent postural-perceptual dizziness (voxel *P* < 0.01, cluster size > 10).

Peak brain regions	Peak coordinates			*T* value	Cluster size
	*X*	*Y*	*Z*		
Cerebelum_8_R (aal)	18	−66	−54	3.2766	17
Vermis_9 (aal)	6	−60	−39	4.2328	28
Cerebelum_10_R (aal)	21	−42	−42	3.7564	11
ParaHippocampal_L (aal)	−21	−24	−27	4.0341	18
Cerebelum_3_L (aal)	−3	−48	−21	3.5265	28
Temporal_Sup_R (aal)	45	−42	12	3.9064	28
Cingulum_Ant_R (aal)	6	36	9	4.0133	16
Occipital_Mid_L (aal)	−33	−72	18	4.0332	10
Caudate_L (aal)	−18	9	21	3.8577	12
Temporal_Inf_L (aal)	−42	−21	−18	4.2294	26
Temporal_Sup_L (aal)	−57	0	−9	4.2845	28
Rectus_R (aal)	18	15	−15	3.0446	14
Frontal_Inf_Oper_L (aal)	−45	12	0	3.4907	22
Temporal_Mid_L (aal)	−66	−39	0	3.2188	11
Frontal_Inf_Tri_L (aal)	−27	12	9	4.7884	42
Precentral_R (aal)	63	9	18	3.9556	37
SupraMarginal_R (aal)	66	−33	27	3.4666	19
Postcentral_L (aal)	−51	−12	30	3.3414	16
Precentral_L (aal)	−42	3	42	3.9337	16
Supp_Motor_Area_L (aal)	0	15	51	4.3512	88
Parietal_Sup_R (aal)	33	−60	54	3.1202	11
Frontal_Sup_L (aal)	−30	−3	66	3.7765	15

All refers to the automated anatomical labeling atlas, which was used to define the brain region labels. Peak coordinates are reported in Montreal Neurological Institute (MNI) space. *X, Y*, and *Z* indicate the three coordinate axes in standard space. L, left; R, right.

In contrast, increased fALFF values were primarily distributed in the temporal, frontal, and parietal regions. These increases involved the left superior temporal gyrus, middle temporal gyrus, and inferior temporal gyrus; the right rectus gyrus and precentral gyrus; the left opercular and triangular parts of the inferior frontal gyrus, precentral gyrus, supplementary motor area, and superior frontal gyrus; as well as the left postcentral gyrus and the right supramarginal gyrus and superior parietal lobule (see Table 5, Figure 3).

### 3.5 Functional connectivity (FC) results

The left insula, right anterior cingulate cortex, and left precuneus were selected as seed regions for whole-brain functional connectivity analyses. All three seed regions were defined based on the corresponding regions in the Automated Anatomical Labeling (AAL) atlas (13). Specifically, the left insula seed was defined as Insula_L, with a central voxel coordinate of *x* = 55, *y* = 133, *z* = 75; the right anterior cingulate cortex seed was defined as Cingulum_Ant_R, with a central voxel coordinate of *x* = 98, *y* = 163, *z* = 88; and the left precuneus seed was defined as Precuneus_L, with a central voxel coordinate of *x* = 83, *y* = 70, *z* = 120. These coordinates were used only for defining the seed ROIs and were not significant peak coordinates derived from the between-group statistical analyses. The use of AAL atlas-based seed definitions helps improve the anatomical standardization and reproducibility of ROI selection and facilitates future validation in larger samples or independent cohorts.

**Figure 4:**
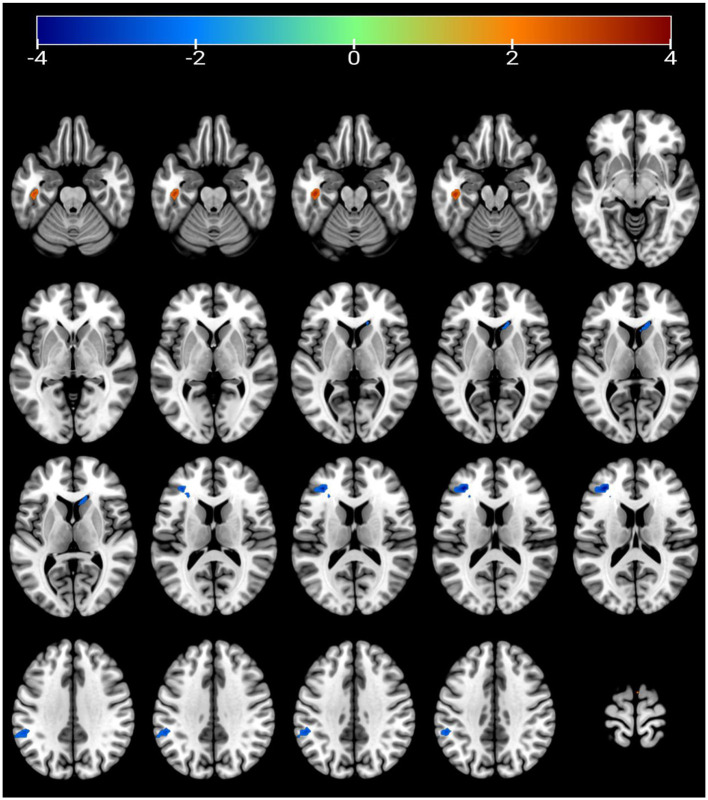
Whole-brain FC differences between the PPPD and HC groups using the left insula as the seed region (Red–yellow regions indicate areas with positive differences in voxel-wise *t*-test comparisons between the two groups, whereas blue–green regions indicate areas with negative differences. The background image is based on the MNI152 template. The color bar represents *T* values, with larger absolute values indicating more significant differences).

When the left insula was used as the seed region, the PPPD group showed decreased functional connectivity with the right caudate nucleus, left middle frontal gyrus, and left inferior parietal lobule compared with the HC group, while increased functional connectivity was observed with the left inferior temporal gyrus and right supplementary motor area (Table 6, Figure 4).

**Table 6:** Results of two-sample *t*-tests for left insula-seeded functional connectivity between the healthy control and persistent postural-perceptual dizziness groups (voxel *P* < 0.01, cluster size > 10).

Peak brain regions	Peak coordinates			*T* value	Cluster size
	*X*	*Y*	*Z*		
Caudate_R (aal)	15	27	6	3.3172	11
Frontal_Mid_L (aal)	−33	36	15	4.1121	31
Parietal_Inf_L (aal)	−51	−39	36	3.2027	35
Temporal_Inf_L (aal)	−42	−21	−21	3.6987	28
Supp_Motor_Area_R (aal)	3	6	75	2.9908	10

All refers to the automated anatomical labeling atlas, which was used to define the brain region labels. Peak coordinates are reported in Montreal Neurological Institute (MNI) space. *X, Y*, and *Z* indicate the three coordinate axes in standard space. L, left; R, right.

**Figure 5:**
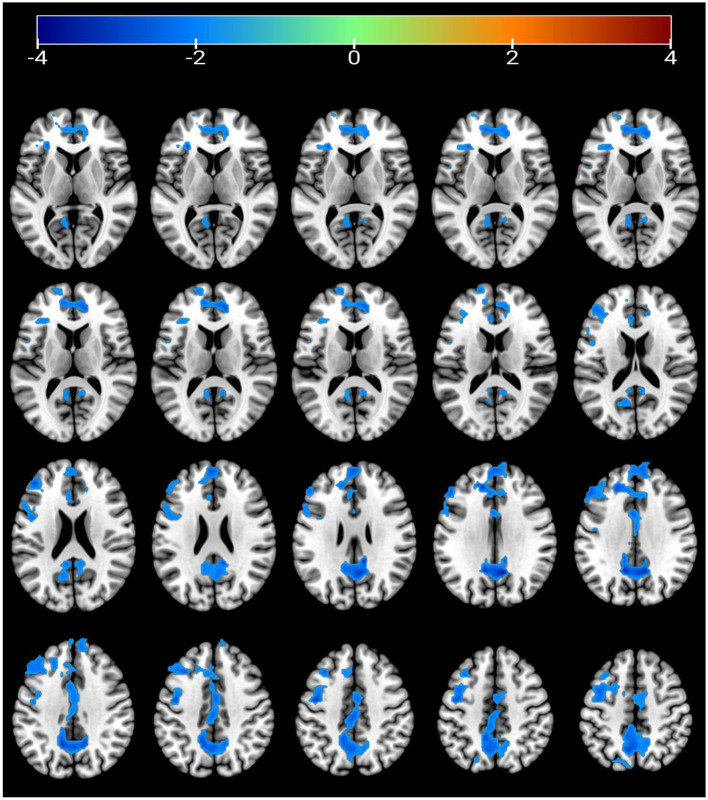
Whole-brain FC differences between the PPPD and HC groups using the right anterior cingulate cortex as the seed region (Red–yellow regions indicate areas with positive differences in voxel-wise *t*-test comparisons between the two groups, whereas blue–green regions indicate areas with negative differences. The background image is based on the MNI152 template. The color bar represents *T* values, with larger absolute values indicating more significant differences).

When the right anterior cingulate cortex was used as the seed region, the PPPD group showed decreased functional connectivity with the right posterior cingulate cortex compared with the HC group (Table 7, Figure 5).

**Table 7:** Results of two-sample *t*-tests for right anterior cingulate cortex-seeded functional connectivity between the healthy control and persistent postural-perceptual dizziness groups (voxel *P* < 0.05, cluster *P* < 0.05, FWE corrected).

Peak brain regions	Peak coordinates			*T* value	Cluster size
	*X*	*Y*	*Z*		
Cingulum_Post_R (aal)	12	−42	27	3.6038	2,338

All refers to the automated anatomical labeling atlas, which was used to define the brain region labels. Peak coordinates are reported in Montreal Neurological Institute (MNI) space. *X, Y*, and *Z* indicate the three coordinate axes in standard space. L, left; R, right.

**Figure 6:**
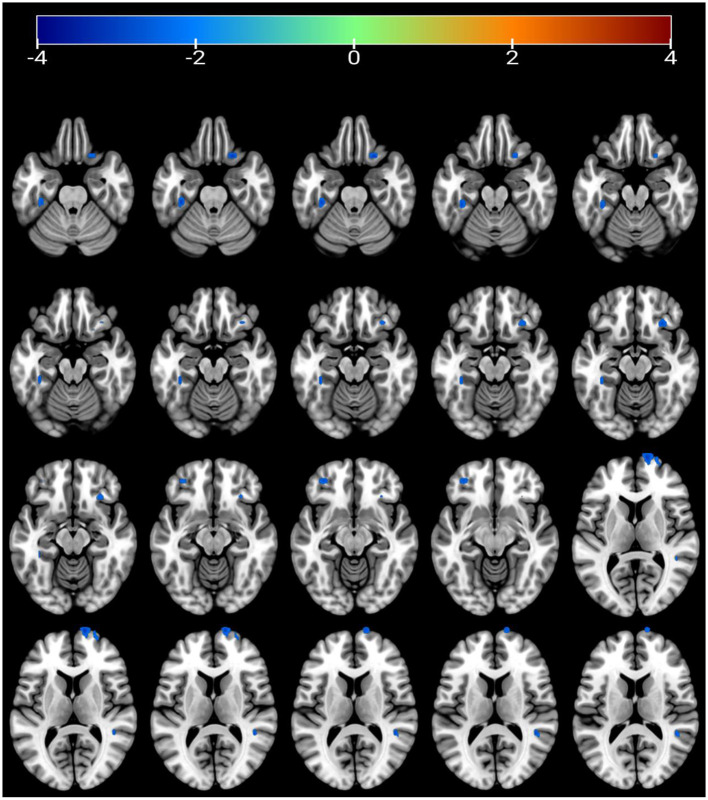
Whole-brain FC differences between the PPPD and HC groups using the left precuneus as the seed region (Red–yellow regions indicate areas with positive differences in voxel-wise *t*-test comparisons between the two groups, whereas blue–green regions indicate areas with negative differences. The background image is based on the MNI152 template. The color bar represents *T* values, with larger absolute values indicating more significant differences).

When the left precuneus was used as the seed region, the PPPD group showed decreased functional connectivity with the left fusiform gyrus, right orbital part of the inferior frontal gyrus, left orbital part of the middle frontal gyrus, right medial superior frontal gyrus, and right superior temporal gyrus compared with the HC group (Table 8, Figure 6).

**Table 8:** Results of two-sample *t*-tests for left precuneus-seeded functional connectivity between the healthy control and persistent postural-perceptual dizziness groups (voxel *P* < 0.01, cluster size > 10).

Peak brain regions	Peak coordinates			*T* value	Cluster size
	*X*	*Y*	*Z*		
Fusiform_L (aal)	−36	−36	−18	3.3612	21
Frontal_Inf_Orb_R (aal)	21	18	−24	3.5488	10
Frontal_Inf_Orb_R (aal)	30	27	−15	2.896	10
Frontal_Mid_Orb_L (aal)	−33	45	−12	2.9463	11
Frontal_Sup_Medial_R (aal)	12	69	9	3.2108	59
Temporal_Sup_R (aal)	45	−42	12	3.5089	11

All refers to the automated anatomical labeling atlas, which was used to define the brain region labels. Peak coordinates are reported in Montreal Neurological Institute (MNI) space. *X, Y*, and *Z* indicate the three coordinate axes in standard space. L, left; R, right.

### 3.6 Clinical-imaging correlation analysis results

To further evaluate the relationship between clinical indicators and brain functional abnormalities in patients with PPPD, clinical-imaging correlation analyses were performed in 21 patients with PPPD. Specifically, abnormal imaging metric values for each PPPD patient were matched individually with the corresponding clinical indicators, and correlations were analyzed between imaging metrics and disease duration, DHI scores, SAS scores, SDS scores, and MoCA scores. Prior to the analysis, normality tests were performed for all variables. Pearson correlation analysis was used for variables that conformed to a normal distribution, whereas Spearman rank correlation analysis was used for variables that did not conform to a normal distribution. Statistical significance was set at *P* < 0.05.

The results showed that disease duration in patients with PPPD was significantly positively correlated with fALFF values in the right superior parietal lobule (*r* = 0.5718, *p* = 0.0084).

DHI scores were significantly positively correlated with fALFF values in the left middle occipital gyrus (*r* = 0.560, *P* = 0.010), and significantly negatively correlated with fALFF values in the left supplementary motor area (*r* = −0.449, *P* = 0.047) and the right superior parietal lobule (*r* = −0.519, *P* = 0.019).

SAS scores were significantly positively correlated with ReHo values in the right middle frontal gyrus (*r* = 0.466, *P* = 0.039), ALFF values in the left Cerebellum_4_5 (*r* = 0.529, *P* = 0.017), and fALFF values in the left middle occipital gyrus (*r* = 0.694, *P* < 0.001). Meanwhile, SAS scores were significantly negatively correlated with fALFF values in the left supplementary motor area (*r* = −0.502, *P* = 0.024) and the right superior parietal lobule (*r* = −0.464, *P* = 0.039).

SDS scores showed a negative correlation trend with fALFF values in the right superior temporal gyrus, but this correlation did not reach statistical significance (*r* = −0.327, *P* = 0.159).

MoCA scores were significantly positively correlated with ReHo values in the left cuneus (*r* = 0.504, *P* = 0.024) and the left precuneus (*r* = 0.538, *P* = 0.014), and significantly negatively correlated with ReHo values in the right medial superior frontal gyrus (*r* = −0.613, *P* = 0.004) and ALFF values in the left Cerebellum_4_5 (*r* = −0.486, *P* = 0.030).

These results suggest that disease duration, symptom severity, anxiety level, and cognitive performance in patients with PPPD are associated, to some extent, with functional abnormalities in brain regions related to visuospatial processing, postural and motor regulation, the cerebellum, and frontoparietal areas.

## 4 Discussion

### 4.1 Abnormalities in the vestibular–cerebellar network

The study found significant functional impairments in the vestibular–cerebellar network of PPPD patients, focusing on various cerebellar subregions. ALFF analysis showed reduced low-frequency amplitude in the left Cerebellum_4_5, Crus II, and right Cerebellum_4_5. fALFF analysis confirmed decreased fractional low-frequency amplitude in the right Cerebellum_8 and 10, left Cerebellum_3, and vermis 9. ReHo analysis revealed reduced local homogeneity in the right Cerebellum_8, 9, and 6. The consistent reductions observed across these metrics suggest compromised central processing and integration of vestibular and postural signals within the cerebellum, aligning with the clinical observation that symptoms are exacerbated during postural transitions or movement. Moreover, a significant increase in ReHo was detected in the left Cerebellum_4_5 region, potentially indicating a compensatory mechanism within the cerebellum. Further analysis also revealed that SAS scores were significantly positively correlated with ALFF values in the left Cerebellum_4_5 whereas MoCA scores were significantly negatively correlated with ALFF values in the same region. These findings suggest that stronger spontaneous neural activity in the left Cerebellum_4_5 is associated with higher levels of anxiety and poorer cognitive performance in patients. This indicates that cerebellar functional alterations are not only related to posture and balance regulation, but may also be involved in the shared neural basis underlying increased anxiety and cognitive decline in patients with PPPD. Taken together with the findings of decreased ALFF but increased ReHo in the left Cerebellum_4_5, these results suggest that cerebellar abnormalities in PPPD may not simply reflect functional hypoactivity. Instead, they may be characterized by reduced spontaneous activity intensity together with abnormally enhanced local synchronization, reflecting complex functional reorganization of the cerebellum after long-term failure of vestibular-postural adaptation.

Previous research has extensively acknowledged the pivotal role of the cerebellum in vestibular and postural control, identifying cerebellar dysfunction as a significant contributor to impaired postural readaptation (14, 15). The cerebellum is integral to the integration of multisensory inputs, particularly in the maintenance of posture and balance. It has been demonstrated that the cerebellum ensures postural stability in dynamic environments by converting sensory information into precise motor commands (16). Furthermore, cerebellar injury can result in a generalized impairment of motor prediction, adversely affecting the accuracy of proprioceptive processing, especially during active movement (17). Previous studies have also documented alterations in cerebellar networks. In a structural analysis, Wurthmann et al. employed voxel-based morphometry and identified reduced gray matter volume in the cerebellum of patients with persistent postural-perceptual dizziness (PPPD) (18). Our finding of decreased functional activity in the cerebellum is consistent with this observation, suggesting that the cerebellum may exhibit not only structural degeneration but also functional hypoactivity. In a SPECT study, Im et al. reported increased bilateral cerebellar perfusion in patients with PPPD, which may reflect a compensatory response to postural control and multisensory information processing deficits (10). Similarly, Van Ombergen et al., observed enhanced connectivity between the cerebellum and the visual cortex/precuneus in patients with visually induced dizziness (19). In addition, recent studies have increasingly recognized that the cerebellum is not only involved in motor control, but also participates in cognitive function and emotional regulation through neural circuits connected with the prefrontal cortex, limbic system, and related cortical regions (20–22). In the present study, ALFF values in the left Cerebellum_4_5 were positively correlated with SAS scores and negatively correlated with MoCA scores, suggesting that abnormal functional activity in this region may be associated with increased anxiety levels and poorer cognitive performance in patients with PPPD. Considering that patients with PPPD are chronically exposed to postural instability, symptom vigilance, and discomfort in spatial orientation, cerebellar functional reorganization may reflect the interaction among enhanced emotional vigilance, impaired allocation of cognitive resources, and increased postural control load in the context of persistent failure of vestibular–postural adaptation. However, because the present study used a cross-sectional correlational design, these findings cannot establish causal relationships and require further validation in future longitudinal and multimodal neuroimaging studies.

In summary, this study found functional abnormalities in multiple cerebellar subregions in patients with PPPD, and functional activity in the left Cerebellum_4_5 was closely associated with anxiety and cognitive performance. Together with previous findings from structural, metabolic, and functional connectivity studies, these results suggest that impaired integration of the vestibular–cerebellar network may not only serve as an important basis for persistent postural instability and motion-induced symptoms in PPPD, but may also contribute to the development of emotional and cognitive symptoms. Future studies should further clarify the specific role of the cerebellum in vestibular–postural regulation, emotional processing, and cognitive control in PPPD, with the aim of providing new insights for diagnostic assessment and targeted intervention.

### 4.2 Abnormalities in the visuospatial processing network

The visual network is mainly composed of the primary visual cortex in the occipital lobe, along with areas like the lingual gyrus, middle occipital gyrus, cuneus, precuneus, and fusiform gyrus. These regions are crucial for visual perception, spatial orientation, shape recognition, and integrating visual input with other sensory information (23–25). In the current study, we observed that the visual network in patients with persistent postural-perceptual dizziness (PPPD) demonstrated functional imbalance, particularly affecting the occipital visual cortex and visual association areas. Analyses of amplitude of low-frequency fluctuation (ALFF) and fractional ALFF (fALFF) revealed significantly decreased low-frequency amplitudes in the right lingual gyrus and left middle occipital gyrus, indicating diminished spontaneous neural activity in these regions. ReHo analysis revealed significantly increased local homogeneity in the left cuneus, indicating compensatory enhancement of local functional synchronization. At the same time, functional connectivity between the left precuneus and the left fusiform gyrus, right orbital part of the inferior frontal gyrus, left orbital part of the middle frontal gyrus, right medial superior frontal gyrus, and right superior temporal gyrus was reduced, reflecting impaired integration among the core nodes of visuospatial processing, visual association regions, prefrontal regulatory areas, and certain vestibular-related regions such as the superior temporal gyrus. These findings suggest a pattern of imbalance in visual information processing characterized by “low activity, enhanced local synchronization, and weakened connectivity,” which may constitute the neural basis for excessive visual dependence and abnormal postural-spatial perception in patients with PPPD.

These findings are highly consistent with previous neuroimaging studies. In terms of structural evidence, Nigro et al. (26) used surface-based morphometry and found reduced cortical folding indices in the right lingual gyrus and occipital extrastriate cortex in patients with PPPD, and these changes were positively correlated with dizziness severity, consistent with the decreased ALFF/fALFF observed in the lingual gyrus in the present study. This suggests that structural and functional abnormalities in this region are closely related to symptom severity (26). In terms of resting-state functional evidence, a series of studies by Li et al. (11, 27, 28) reported reduced spontaneous activity in the right precuneus and cuneus, as well as weakened functional connectivity with the precentral gyrus and premotor cortex, along with reduced within-network connectivity of the precuneus (27). Although we observed increased ReHo in the left cuneus, the widespread decrease in FC between the left precuneus and multiple regions was highly consistent with these previous findings, all pointing to dysfunction of the cuneus/precuneus as a hub for visual-spatial integration. In addition, Riccelli et al. (29) found marked enhancement of visual cortical activity during vertical motion stimulation (29), and Passamonti et al. (30) reported increased activity of the inferior frontal gyrus and enhanced connectivity with bilateral visual areas during visual stimulation in PPPD patients with high neuroticism, suggesting that the reduced connectivity between the fusiform gyrus observed in our study may represent a manifestation of network fatigue after long-term excessive visual compensation (30). At the level of emotional regulation, a resting-state study by Lee et al. (31) showed that anxiety was a key driver of widespread connectivity changes across the occipital, frontal, and parietal lobes, and that the degree of anxiety was positively correlated with connectivity between the visual cortex and frontal regions (31). In contrast, our study found reduced connectivity between the precuneus, a key node of the default mode network in the parietal midline, and the orbitofrontal and medial superior frontal regions. Together, these findings present a functionally complementary pattern: the former reflects the promotion of excessive information input from the primary and higher-order visual cortices to the prefrontal cortex by anxiety, whereas the latter reveals weakened functional integration between the precuneus and emotion-regulating regions such as the orbitofrontal cortex. This shared pattern suggests that emotional comorbidity may exacerbate visual network imbalance through multiple pathways.

Further correlation analyses between clinical indicators and imaging data showed that DHI scores were significantly positively correlated with fALFF values in the left middle occipital gyrus, and SAS scores were also significantly positively correlated with fALFF values in the left middle occipital gyrus, suggesting that changes in spontaneous neural activity in visual processing regions are closely associated with patients' subjective dizziness-related handicap and anxiety severity. This finding indicates that visual network abnormalities in patients with PPPD may not only reflect impaired visual–vestibular integration, but may also be related to excessive reliance on visual information, heightened sensitivity to visual stimuli, and an anxiety-related state of sensory vigilance. Notably, MoCA scores were significantly positively correlated with ReHo values in the left cuneus and left precuneus, suggesting that local synchronization within visuospatial integration nodes is closely related to cognitive performance. Given the role of the precuneus in visuospatial orientation, multisensory integration, and cognitive regulation (32), this finding suggests that enhanced local coherence in this region may have a certain cognitive supportive or compensatory significance. When the regulatory efficiency of this visuospatial integration network is insufficient, patients may be more prone to spatial disorientation, aggravated dizziness, and increased cognitive load in complex visual environments. Therefore, symptom exacerbation in complex visual environments among patients with PPPD may not simply arise from abnormal visual–vestibular integration, but may be closely related to abnormal coupling among visual processing, emotional vigilance, and cognitive regulation.

In summary, this study found widespread functional abnormalities in the visuospatial processing network of patients with PPPD, characterized by decreased spontaneous activity in occipital visual processing regions, enhanced local synchronization in the cuneus, and weakened functional connectivity between the precuneus and brain regions including visual areas, the prefrontal cortex, and the superior temporal gyrus. Further clinical correlation analyses showed that fALFF values in the left middle occipital gyrus were associated with dizziness-related handicap and anxiety severity, whereas ReHo values in the left cuneus and precuneus were associated with cognitive performance. These findings suggest that abnormalities in the visuospatial processing network may constitute an important neural basis for visual dependence, symptom exacerbation in complex visual environments, increased anxiety levels, and altered cognitive performance in patients with PPPD. Visual network abnormalities in PPPD do not represent a single visual processing disorder, but may instead reflect network-level alterations involving the combined imbalance of visual–vestibular integration, emotional vigilance, and cognitive regulation.

### 4.3 Abnormalities in the postural control and motor regulation network

Previous studies have identified structural abnormalities in brain regions related to motor control in patients with PPPD. Structural neuroimaging has shown reduced gray matter volume in sensorimotor-related regions, including the precentral gyrus, supplementary motor area (SMA), and cerebellum. Notably, the gray matter volume of the SMA was negatively correlated with disease duration, suggesting that these key regions involved in motor planning and postural regulation may undergo structural degeneration or reorganization during the chronic course of the disorder (10). Building on these findings, the present study further provided functional evidence at the regional level. ReHo analysis revealed significantly increased local synchronization in the right precentral gyrus and SMA, while ALFF and fALFF analyses also indicated increased low-frequency amplitude in the precentral gyrus, suggesting enhanced spontaneous neural activity and stronger local neuronal coordination in these regions at rest. In neuroimaging studies, a pattern of reduced structure but increased functional activity is not uncommon and is generally considered to reflect compensatory functional reorganization. When structural integrity declines or network efficiency decreases, the brain may maintain functional output by increasing local neural synchrony or metabolic activity (33). Therefore, the increased ReHo and low-frequency amplitude observed in the present study may indicate compensatory enhancement of metabolic or neural activity in the precentral gyrus and SMA in patients with PPPD, thereby offsetting the reduced efficiency of information integration during postural control and motor planning. In addition, the clinical correlation analysis showed a significant positive correlation between disease duration and fALFF values in the right superior parietal lobule. The right superior parietal lobule is an important region involved in spatial attention, postural perception, and multisensory integration (34). Therefore, this finding suggests that patients with longer disease duration may exhibit stronger spontaneous activity in parietal regions related to spatial information processing and postural perceptual regulation. More importantly, this result is relevant to the question of whether the observed brain functional abnormalities represent pre-existing vulnerability factors or changes that emerge during the course of PPPD. The positive association between disease duration and right superior parietal activity may indicate that prolonged persistent dizziness is accompanied by progressively increased reliance on parietal regions responsible for spatial and postural integration. This pattern may reflect an adaptive or maladaptive compensatory response to long-term unreliable vestibular input and persistent dizziness-related symptoms, rather than a purely pre-existing neural characteristic. Meanwhile, DHI scores were significantly negatively correlated with fALFF values in the left SMA, and SAS scores were significantly negatively correlated with fALFF values in both the left SMA and the right superior parietal lobule. The SMA is involved in motor preparation, postural regulation, and motor control, whereas the superior parietal lobule participates in spatial orientation and multisensory integration. These findings suggest that although prolonged disease duration may be accompanied by enhanced functional activity in parietal regions involved in spatial integration, spontaneous activity in the SMA and parietal regions may instead decrease in patients with greater dizziness-related handicap or higher anxiety levels. This indicates that such functional enhancement may not be sufficient to stably maintain postural control and spatial integration. In particular, elevated anxiety levels may simultaneously weaken functional activity in regions involved in motor preparation and spatial integration, thereby further aggravating dizziness, unsteadiness, and motion-induced symptoms. Meanwhile, DHI scores were significantly negatively correlated with fALFF values in the left SMA, and SAS scores were significantly negatively correlated with fALFF values in both the left SMA and the right superior parietal lobule. The SMA is involved in motor preparation, postural regulation, and motor control, whereas the superior parietal lobule participates in spatial orientation and multisensory integration. These findings suggest that although prolonged disease duration may be accompanied by enhanced functional activity in parietal regions involved in spatial integration, spontaneous activity in the SMA and parietal regions may instead decrease in patients with greater dizziness-related handicap or higher anxiety levels. This indicates that such functional enhancement may not be sufficient to stably maintain postural control and spatial integration. In particular, elevated anxiety levels may simultaneously weaken functional activity in regions involved in motor preparation and spatial integration, thereby further aggravating dizziness, unsteadiness, and motion-induced symptoms.

In addition, we found significantly increased functional connectivity between the left insula and the right SMA. The insula is regarded as an important hub for multisensory integration and interoceptive processing, and it plays a key role in vestibular, somatosensory, and autonomic regulation (35, 36, 52). Increased insula–SMA connectivity may reflect strengthened transmission of sensory information to the motor control system, thereby facilitating postural regulation and motor preparation. However, such enhancement may also represent a low-efficiency compensatory network reorganization. When visual–vestibular or sensorimotor integration pathways are disrupted, the brain may attempt to preserve balance control by strengthening connectivity between key nodes; nevertheless, this compensation is often accompanied by reduced regulatory efficiency at the network level, which may ultimately manifest as persistent dizziness, unsteadiness, and exacerbation of movement-induced symptoms (37).

In summary, the present study found marked functional abnormalities in the postural control and motor regulation networks of patients with PPPD. These abnormalities were mainly characterized by increased local functional synchronization and spontaneous neural activity in the precentral gyrus and supplementary motor area (SMA), as well as enhanced functional connectivity between the insula and SMA. These changes suggest that patients with PPPD may enhance local activity and network connectivity in sensorimotor-related brain regions to maintain postural regulation and motor preparation.

Further clinical correlation analyses showed that patients with longer disease duration exhibited increased functional activity in the right superior parietal lobule, suggesting that persistent long-term symptoms may increase patients' reliance on spatial attention, postural perception, and multisensory integration. In contrast, patients with greater dizziness-related handicap showed decreased functional activity in the left SMA, while patients with higher anxiety levels showed reduced functional activity in both the left SMA and right superior parietal lobule. These findings indicate that symptom burden, particularly increased anxiety levels, may be associated with reduced regulatory capacity of networks involved in postural control, motor preparation, and spatial integration.

Taken together, abnormalities in the postural control and motor regulation networks in patients with PPPD do not simply reflect functional enhancement or reduction. Instead, they may involve the coexistence of disease duration-related adaptive functional enhancement and symptom burden-related decline in regulatory capacity. This functional imbalance may constitute an important neural basis for the persistence of chronic dizziness, unsteadiness, and motion-induced symptoms in patients with PPPD.

### 4.4 Abnormalities in the emotion and salience regulation network

The pathophysiological mechanisms of PPPD involve not only abnormalities in multisensory vestibular–visual integration, but are also closely associated with disturbances in the salience network and emotion-regulation network. Previous neuroimaging studies have provided supporting evidence from both structural and functional perspectives. In terms of structural findings, Wurthmann et al. used voxel-based morphometry and found significantly reduced gray matter volume in the cingulate cortex and caudate nucleus of patients with PPPD, while the gray matter volume of the precentral gyrus and supplementary motor area decreased with longer disease duration (38); Popp et al. observed an increase in gray matter volume in frontal regions in patients with precursor disorders of PPPD, and this increase was positively correlated with depression scores, suggesting that the prefrontal cortex–cingulate cortex-insula hub may aggravate postural imbalance through excessive self-monitoring (39). Functional studies further confirmed these abnormalities. Using task-based fMRI with sound-induced vestibular stimulation, Indovina et al. found reduced activity in the insula and cingulate cortex in patients with chronic subjective dizziness, together with decreased connectivity with the superior temporal gyrus, hippocampus, and other regions (40); A SPECT study by Na et al. also showed reduced perfusion in the left posterior insula in patients with PPPD (10). Although these findings suggest abnormal activity and connectivity in the insula, anterior cingulate cortex, and caudate nucleus, most of the evidence is limited to single-modality analyses and therefore cannot fully characterize the dynamic local and global network alterations present at rest.

In the present study, multimodal resting-state fMRI analyses further clarified the characteristic functional abnormalities of the salience network and emotion-regulation network in patients with PPPD. The results showed significantly increased ReHo in the left insula, suggesting compensatory enhancement of local synchronization in this region at rest. In contrast, both ALFF and fALFF in the right anterior cingulate cortex were significantly decreased, indicating reduced spontaneous neural activity. Functional connectivity analysis using the left insula as the seed region revealed significantly weakened connectivity with the caudate nucleus, middle frontal gyrus, and inferior parietal lobe, but significantly strengthened connectivity with the inferior temporal gyrus and supplementary motor area. Meanwhile, when the right anterior cingulate cortex was used as the seed region, its connectivity with the posterior cingulate cortex was also significantly reduced. These findings are highly consistent with previous structural evidence, including gray matter reduction in the cingulate cortex and caudate nucleus, as well as functional evidence of reduced insula-cingulate activity and connectivity. At the same time, the present multimodal resting-state analysis further complements these observations by revealing more detailed network features, including local compensatory enhancement in the insula and a specific disruption of connectivity among the insula, caudate nucleus, anterior cingulate cortex, and posterior cingulate cortex.

Further clinical correlation analyses showed that SAS scores were significantly positively correlated with ReHo values in the right middle frontal gyrus, ALFF values in the left Cerebellum_4_5 and fALFF values in the left middle occipital gyrus, while they were significantly negatively correlated with fALFF values in the left supplementary motor area and right superior parietal lobule. These findings indicate that increased anxiety levels may be accompanied by enhanced activity in prefrontal regulatory regions, visual processing areas, and cerebellar nodes, as well as reduced activity in regions involved in motor preparation and spatial integration. This pattern suggests that anxiety may not merely be a concomitant symptom, but may further contribute to the persistence of PPPD symptoms by enhancing visual vigilance and interoceptive monitoring while weakening the efficiency of postural-motor regulation. In contrast, SDS scores showed only a negative trend-level correlation with fALFF values in the right superior temporal gyrus, without reaching statistical significance, suggesting that anxiety-related mechanisms may be more prominent than depressive factors in the present sample.

In summary, the present study found marked functional abnormalities in the emotional and salience regulation networks of patients with PPPD. These abnormalities were mainly characterized by increased local coherence in the left insula, decreased spontaneous neural activity in the right anterior cingulate cortex, weakened connectivity between the insula and the caudate nucleus, middle frontal gyrus, and inferior parietal lobule, enhanced connectivity between the insula and the inferior temporal gyrus and SMA, and reduced connectivity between the anterior cingulate cortex and posterior cingulate cortex. These findings suggest that patients with PPPD may exhibit enhanced insular interoceptive signal processing, reduced cingulate-mediated emotional-attentional regulation, and imbalanced connectivity between the salience network and frontoparietal regulatory and motor control networks. Further clinical correlation analyses showed that increased anxiety levels were associated with enhanced activity in prefrontal regulatory regions, visual processing areas, and cerebellar nodes, as well as reduced activity in motor preparation and spatial integration regions such as the SMA and superior parietal lobule. These findings suggest that anxiety may not simply bean accompanying emotional symptom, but may participate in the persistent maintenance of PPPD symptoms by enhancing visual vigilance and interoceptive monitoring while weakening postural-motor and spatial integration regulation. In contrast, depressive scores showed only trend-level associations with activity in relevant brain regions and did not reach statistical significance, suggesting that anxiety-related neural mechanisms may be more prominent than depressive factors in the present sample. Overall, emotional regulation abnormalities in PPPD may reflect a network imbalance pattern characterized by “enhanced interoceptive monitoring—reduced emotional-attentional regulation—restricted postural-motor regulation,” which may be closely related to persistent dizziness, unsteadiness, and symptom chronicity.

### 4.5 Default mode network (DMN)

The default mode network (DMN) is a set of brain regions that exhibit coherent activity and a high level of metabolic activity during the resting state (41). It primarily consists of core nodes including the posterior cingulate cortex/precuneus (PCC/precuneus), medial prefrontal cortex (mPFC), and bilateral inferior parietal lobules (IPL) (42, 43). The DMN is closely associated with a variety of higher-order cognitive processes, including self-referential thinking, retrieval and integration of episodic memory, future planning, and internal simulation (44, 45). It is considered a core network for internal mental activity and the maintenance and construction of the “self.” During tasks requiring externally directed attention, DMN activity is typically suppressed (43). Abnormal functional connectivity within the DMN has been widely reported in various neurological and psychiatric disorders, such as Alzheimer's disease, depression, and schizophrenia (46, 47). Several independent studies using functional connectivity analysis have demonstrated significantly reduced connectivity within the posterior DMN (pDMN) in patients with PPPD. A key study further showed that, compared with healthy controls, PPPD patients exhibited significantly decreased functional connectivity between the precuneus and bilateral precuneus regions (28).

Building on these findings, the present study further demonstrated that patients with PPPD showed significantly reduced activity in the right anterior cingulate cortex, whereas both local activity and local synchronization in the left precuneus were significantly increased. Seed-based FC analysis revealed decreased connectivity between the anterior cingulate cortex and posterior cingulate cortex, as well as reduced connectivity between the precuneus and the right superior frontal gyrus and medial superior frontal gyrus (*F* = 3.2108). Taken together, previous studies have consistently shown that the DMN in PPPD patients—particularly the posterior DMN centered on the PCC/precuneus—exhibits significantly weakened internal functional connectivity, suggesting impaired roles in maintaining internal spatial representations and integrating self-related states. The present findings provide further evidence to refine this model. On the one hand, the reduced connectivity between the anterior and posterior cingulate cortex reinforces the notion of disrupted anterior–posterior integration within the DMN as a stable feature. On the other hand, we observed increased local activity and synchronization in the precuneus, accompanied by decreased connectivity with the superior frontal gyrus and medial superior frontal gyrus. This pattern suggests that the precuneus is not simply “hypofunctional,” but rather may exist in a state of local hyperactivity with constrained network-level output.

From a therapeutic perspective, the widespread network-level abnormalities observed in the present study suggest that PPPD may require a multidimensional treatment strategy rather than a single intervention targeting vestibular symptoms alone. Abnormalities in vestibular–cerebellar and postural–motor regions support the continued use of individualized vestibular rehabilitation, balance training, and postural control exercises to promote adaptive recalibration of vestibular and motor responses. Functional alterations in visual and visuospatial processing regions indicate the potential value of graded exposure to complex visual environments and visual-motion desensitization, particularly in patients whose symptoms are exacerbated by visual stimuli. In addition, the involvement of the insula, anterior cingulate cortex, prefrontal regions, and default mode network suggests that interventions targeting anxiety, depressive symptoms, heightened bodily vigilance, and maladaptive excessive attention to bodily discomfort may also be essential. Therefore, future treatment studies should design integrated intervention protocols that combine vestibular rehabilitation, visual desensitization, postural–motor training, and psychological or pharmacological interventions when clinically appropriate. Further longitudinal neuroimaging studies are needed to determine whether these interventions can normalize abnormal brain activity and connectivity, and whether such neural changes correspond to improvements in clinical symptoms.

### 4.6 Regional neural differences between persistent postural-perceptual dizziness and mal de débarquement syndrome

Neural overlap and regional specificity between persistent postural-perceptual dizziness and mal de débarquement syndrome (MdDS). A useful comparison can be made with mal de débarquement syndrome, another functional vestibular condition that is sometimes confused with PPPD. Clinically, MdDS is typically characterized by persistent non-spinning oscillatory vertigo, described as rocking, bobbing, or swaying, which begins after exposure to passive motion such as sea, air, or land travel and is temporarily relieved by renewed passive motion, such as driving (48). This clinical pattern differs from PPPD, in which dizziness or unsteadiness is usually aggravated by upright posture, active or passive motion, and complex visual environments (1, 48).

At the neural level, neuroimaging studies of MdDS have identified a relatively distinctive pattern centered on the left entorhinal cortex and amygdala, with hyper metabolism in this limbic–spatial memory region and increased connectivity to posterior visual–vestibular and spatial processing areas, including the primary visual cortex, motion-sensitive visual cortex, superior parietal lobule, and middle temporal gyrus, together with reduced connectivity to prefrontal regions (49, 50). In transient MdDS, metabolic abnormalities have also been reported in the bilateral occipital and prefrontal cortices, accompanied by hypo metabolism in the vestibulocerebellum, particularly the nodulus and uvula, and reduced connectivity between vestibular and visual association regions (51).

These findings partially parallel the present results by suggesting shared involvement of visual–vestibular integration, visuospatial attention, prefrontal regulation, and cerebellar processing. However, the regional distribution of abnormalities differs. In the present study, PPPD was not dominated by an entorhinal cortex and amygdala-centered abnormality. Instead, the main findings involved reduced or reorganized activity in the cerebellar posterior lobe, anterior cingulate cortex, occipital and temporal visual–vestibular regions, together with altered connectivity of the left insula, right anterior cingulate cortex, and left precuneus with frontoparietal, basal ganglia, visual, and default-mode-related regions. Therefore, although the two disorders may share a broader mechanism of maladaptive visual–vestibular and spatial network reweighting, MdDS appears to show a more specific entorhinal–amygdala–posterior visual and spatial memory signature, whereas PPPD is more characterized by distributed dysfunction of cerebellar, salience, default-mode, visuospatial, and postural-motor regulation networks. This distinction supports partial neurobiological overlap but argues against treating the two disorders as interchangeable entities.

## 5 Limitations

Several limitations of this study should be acknowledged. First, the relatively small sample size may have limited the statistical power and generalizability of the findings. Although multiple resting-state functional magnetic resonance imaging metrics were employed to provide convergent evidence, the results should be interpreted with caution and require replication in larger, independent cohorts.

Second, patients with persistent postural-perceptual dizziness exhibited a degree of clinical heterogeneity, including differences in disease duration, dizziness-related disability, emotional symptoms, and cognitive performance. Clinical–imaging correlation analyses were conducted to explore the relationships between these clinical characteristics and brain functional alterations. However, given the limited sample size, these associations should be considered preliminary. Future studies with larger cohorts are warranted to further investigate the influence of clinical heterogeneity through more comprehensive analyses, including covariate-adjusted, subgroup, and longitudinal approaches.

Third, the cross-sectional nature of the study precludes conclusions regarding the causal relationship between the observed brain functional abnormalities and PPPD. It remains unclear whether these alterations represent predisposing factors, consequences of the disorder, or compensatory neural adaptations. Longitudinal studies are needed to elucidate the temporal dynamics of brain functional changes and their relationship with clinical manifestations.

## 6 Conclusions

In summary, by combining amplitude of low-frequency fluctuation, fractional amplitude of low-frequency fluctuation, regional homogeneity, and seed-based functional connectivity analyses, the present study demonstrated widespread resting-state functional abnormalities in patients with PPPD. These abnormalities involved the vestibular–cerebellar network, visuospatial processing network, postural and motor regulation network, emotional and salience regulation network, and default mode-related network. Specifically, patients showed reduced spontaneous activity or local synchronization in regions such as the cerebellar posterior lobe, occipital lobe, temporal lobe, anterior cingulate cortex, and basal ganglia, together with increased activity or local synchronization in parts of the frontal lobe, parietal lobe, insula, parahippocampal region, precuneus, supplementary motor area, and selected cerebellar subregions.

At the network level, altered functional connectivity among the insula, caudate nucleus, prefrontal cortex, inferior parietal lobule, supplementary motor area, anterior cingulate cortex, posterior cingulate cortex, precuneus, fusiform gyrus, and superior temporal gyrus suggests disrupted coordination among salience monitoring, emotional regulation, visual–vestibular integration, postural–motor control, and self-referential cognitive processing systems. Furthermore, exploratory clinical–imaging correlation analyses showed that disease duration, dizziness handicap, anxiety severity, and cognitive performance were associated with functional alterations in the superior parietal lobule, middle occipital gyrus, supplementary motor area, cerebellar lobules IV–V, cuneus, precuneus, and medial superior frontal gyrus.

Overall, these findings suggest that persistent postural-perceptual dizziness is not merely an isolated vestibular disorder, but rather reflects a distributed network disorder characterized by impaired visual–vestibular–postural integration, heightened visual and interoceptive vigilance, abnormal emotional and salience regulation, disrupted self-referential and visuospatial processing, and insufficient postural–motor compensation. The observed clinical–imaging associations further indicate that these neural abnormalities may contribute to symptom persistence, dizziness-related disability, anxiety-related symptom amplification, and cognitive burden in patients with PPPD.

## Data Availability

The data analyzed in this study is subject to the following licenses/restrictions: due to privacy and ethical restrictions, the datasets generated and/or analyzed during the current study are not publicly available. De-identified data may be available from the corresponding authors upon reasonable request and with approval from the institutional ethics committee. Requests to access these datasets should be directed to 17839964863@163.com.
